# Resolution of Respect to the Memory of Dr. E. A. Stebbins

**Published:** 1896-12

**Authors:** 


### Obituary.

### RESOLUTION OF RESPECT TO THE MEMORY OF DR. E. A. STEBBINS.

A committee of the Northeastern Dental Association, consisting of Drs. C. T. Stockwell, George A. Maxfield, of Holyoke, Newton Morgan, Edward S. Gaylord, of New Haven, Conn., and W. H. Jones, of Northampton, reported the following resolution in memory of Dr. E. A. Stebbins, of Shelburne Falls, who died during the year:

Whereas, Every member of the branch of this Association, which constituted the old Connecticut Valley Dental Society, must realize that we meet to-day under the shadow of personal grief. It is scarcely probable, however, that there should be any one present who will not share in some degree in the sense of loss and sorrow that comes to us, as on this occasion especially we recall the fact that Dr. Stebbins is dead. To us of the Connecticut Valley Society there was but one Dr. Stebbins. He needed no other designation in the rich association and experience of a long and eventful past. He needs no other now. No meeting was quite complete if Dr. Stebbins was absent. But who of us can to-day recall a meeting when he was not present from beginning to the end, ready always to bear his portion and more than his portion of whatever burdens there might be to share, and also to contribute of his rich store of wisdom, sound judgment, and experience, freely, modestly, generously, nobly. As a dentist we respected and honored him. As a professional man he stood very near, if, indeed, he did not reach the true type of the ideal. As a man, he was an abiding inspiration. On whatever side we met him his touch was life and evermore life. Unostentatious, courteous, courageous, dignified, cordial, ever helpful in spirit towards all, but as true to his convictions and his sense of right and duty as the rock-ribbed mountains that so gloriously encircle his newly-made grave. We loved him.

*Resolved,* That our secretary be requested to convey to Dr. Stebbins's widow and children the assurance of our sympathy in this common loss and sorrow.

